# Considering tradeoffs in “local” food policies: examples from school feeding programmes

**DOI:** 10.3389/fnut.2023.1242493

**Published:** 2023-09-12

**Authors:** Becca B. R. Jablonski, Paul Milbourne, Siobhan Maderson, Kevin Morgan

**Affiliations:** School of Geography and Planning, University of Cardiff, Cardiff, United Kingdom

**Keywords:** food policy, sustainable development goals, public food procurement, farm to school, local food

## Abstract

City, national, and multinational governments are increasingly leveraging nutrition programme spending, specifically pursuing policies that require or incentive “local” procurement, to meet a myriad of goals. However, these policies involve tradeoffs that are often not fully considered by government officials, planners, and advocates. This perspective article provides some examples of those tradeoffs from the peer-reviewed literature, which, we argue, are useful to consider in setting school feeding programme policies to achieve sustainability goals.

## 1. Introduction

The understanding that food systems play a critical role in achieving multiple United Nations Sustainable Development Goals (UN SDGs) [e.g., (1, 2)], is driving many city, national, and multinational governments to implement sustainable food policies. Leveraging municipal spending on nutrition programmes, with a focus on “local” school feeding programmes, appears to be the most common strategy implemented by governments worldwide [e.g., (3, 4)]. This is likely due to the size of school feeding programme budgets, which are estimated at $70B U.S. annually, reaching about 368 million children globally (5).

Governments are creating policies to leverage this spending in order to achieve multiple SDGs, including reducing hunger and promoting good health and well-being (UN SDGs 2 and 3), creating opportunities for decent work and economic growth (UN SDG 8), and creating or growing markets for sustainably produced food (UN SDG 12) (6). For example, the National School Feeding Programme in Brazil requires 30 percent of its budget be used to purchase food from family farms (2). In the United States (U.S.), a dedicated Federal funding programme supports farm to school programmes, which helped to spur the reported $1.26 billion spent by schools on locally procured items in the 2018–2019 school year (4). Part of governments’ rationale for these policies is that local purchasing are believed to increase consumption of fresh, healthy food, increase farm access to markets, and reduce greenhouse gas emissions and waste through shorter supply chains (6).

However, from a theory of economic policy perspective, multiple policy instruments are needed to meet multiple goals. When a single policy instrument is used to meet multiple goals (or there are fewer instruments than targets), the best one can achieve is a second-best outcome that requires placing some priorities (weights) on the goals [e.g., (7)]. In other words, tradeoffs across goals will inherently exist if multiple policy instruments are not available. In line with this theoretical framework, “local” school feeding programme policies involve tradeoffs, and these are often not fully considered by government officials, planners, and advocates. Further, these policies can fall into a “local trap,” wherein local is assumed to be *better* across many dimensions (8). This perspective article provides some examples of those tradeoffs from the peer-reviewed literature, which, we argue, are useful to consider in setting school feeding programme policies to make progress towards the UN’s SDGs.

## 2. Perspective guided by literature

### 2.1. Reducing hunger and promoting good health and well-being (UN SDGs 2 and 3)

Increasing local food procurement in schools is frequently aligned with governments’ desire to improve healthy eating goals among students and their families, as well as reducing malnutrition and food insecurity [e.g., (2)]. It is well-documented that providing meals in schools, and especially healthy meals, supports positive student educational and health outcomes [e.g., (9, 10)], but additional benefits resulting from local procurement are much less clear. Part of the challenge is that conducting research in school settings is notoriously difficult, and, as a result, there are few peer-reviewed studies that employ rigorous methods (e.g., randomized control trials). Those that do provide a rigorous analysis suggest that local food in schools may improve food and nutrition-related knowledge and encourage healthy food selection among children, but the evidence on increasing fruit and vegetable consumption is mixed (11). In other words, the health effects are unclear if differences in knowledge and selection do not result in changes in consumption.

Tradeoffs may result from the fact that locally grown and raised food may be more expensive than nonlocal items (12). Local supply chains may incur higher transaction costs due to decreased efficiency. This issue may be exacerbated if policies prioritize purchases from local smallholders that have higher costs of production (13). As schools are budget constrained, shifts to local purchasing may involve tradeoffs that could negatively impact other procurement decisions and, thus, student health outcomes (12).

### 2.2. Creating opportunities for decent work and economic growth (UN SDG 8)

The food system is global, concentrated in the hands of major food corporations, and vertically integrated, which enhances its economic efficiency (14). Due to budget constraints, schools use this system to procure most of their local products. In other words, most local food does not move directly from the farm to the school, but through an intermediary, such as a wholesaler or distributor (15). Given the market power of the large-scale supply chain actors—even those that are moving locally grown and raised items—there is insufficient evidence that they pass any price premiums to local farmers. In fact, research on food supply chain consolidation and vertical integration finds economic benefits for consumers, but complex implications for farmers, and negative impacts for smallholders and rural communities (14).

Another potential challenge around “local” strategies centers around the economic concept of “beggar-thy-neighbor” [e.g., (16)]. If there is not an absolute increase in purchasing (and therefore production or the value of production) as a result of changes in school food buying patterns (which is unlikely to be the case other than perhaps in the poorest countries), then local purchases are likely to represent a shift in sourcing. Thus, shifts to local purchasing by schools come at the expense of nonlocal products (17). While there may be a local economic benefit, there may be a disbenefit to other regions. Given that local school food procurement strategies are more dominant in more affluent countries, this strategy may negatively impact the global South and more agriculturally dependent economies (18).

Similarly, as most of the world’s population lives in urban areas (19), and hundreds of cities are enacting food policies [e.g., (20, 21)], gains in urban regions may come at the expense of rural ones. Research from several countries specifically calls for improved regional governance and infrastructure structures to more effectively link urban, peri-urban, and regional areas [e.g., (22, 23)]. As rural stakeholders, and particularly rural farmers, that still represent the majority of food production are often left out of urban food policy dialogues (24), broader opportunities for positive regional impacts from “local” food policies remain limited.

Though not a tradeoff *per se*, a further area of complexity is that “local” food is often produced by nonlocal workers. As the major food retailers have sought to reduce food costs for their consumers, cost pressures have been passed onto food producers and processors. This has led to shifts in local work regimes, involving more precarious working conditions for farm laborers and the increased use of international migrant workers to harvest, process, and package food. Some research claims that the relocalization of global North food systems may have detrimental impacts on the livelihoods of those farmers and agricultural laborers in global South countries reliant on export markets to sell their produce. Indeed, particular sectors of the farming system are now as dependent on migrant labor as they are on climate, soils and fertilizers. Certainly, there has been little appetite amongst national governments to discuss how the structural conditions of agri-food work could be improved to provide decent local employment opportunities. Instead, there appears to be an acceptance that such work will continue to be undertaken by international migrant workers, with the agri-food sector viewed as an exception to restrictive national migration policies across several Global North countries (25).

### 2.3. Responsible production (UN SDG 12)

Many municipalities are in part leveraging school feeding programmes to incentivize transitions to more environmentally sustainable procurement, but there is little evidence to support that producers selling through local markets use more environmentally sound practices. In the U.S., some studies indicate that producers using conventional markets spend more, on average, for chemical inputs and fertilizers than locally oriented operations, which are more likely to use manure (26). However, the overall use of chemical inputs and fertilizers is declining, with the biggest declines occurring on operations using conventional markets. Conventionally oriented producers are also more likely to use no-till or conservation tillage (26). Further, shifts to local food procurement with additional preferences for environmentally friendly labels, such as organic, can result in additional tradeoffs if the specific context of climate, place, and commodity are not considered [e.g., (27)]. For example, in drought-prone, semi-arid cropland systems there can be negative effects on erosion potential and organic carbon in the soil (28).

The link between local foods and food waste is even less clear. An inverse relationship may exist between the amount of food waste and packaging waste if local foods use less processing and packaging, possibly resulting in more food waste from spoilage and less efficient home preparation (26). The limited research in school settings has mixed results, mostly with small sample sizes using plate waste assessments [e.g., (29)]. Further, some research related to community supported agriculture schemes finds that consumers are more likely to waste products that they are unsure how to prepare (30). Given constraints around seasonality in many parts of the world, this challenge may also apply to school settings, where students may be less familiar with seasonal products and thus less willing to try them.

The literature also suggests that the provision of local foods may actually result in a larger transportation footprint in terms of both greenhouse gas emissions and energy consumption due to transportation inefficiencies [e.g., (31–33)]. Although there are thought pieces and limited research that suggest environmental benefits to localized food systems [e.g., (34)], the overwhelming takeaway is that what you eat may be more important than whether or not it is local (32). In other words, local is not inherently environmentally better; impacts need to be carefully considered within the context of many other factors.

## 3. Conclusion

Although there are potential benefits for food policies that view “local” school food procurement as a step towards achieving sustainable development goals, we caution policymakers, advocates, and planners not to make *a priori* assumptions that local is better. Returning to the theory of economic policy, achieving multiple goals will require more policy instruments than just changes to procurement structure (7). If policymakers do not consider what these other instruments might be and, tradeoffs will exist. However, starting with a more holistic view of the food system—both throughout the entire supply chain as well as across jurisdiction – can help policymakers to more carefully consider the multiple policy instruments that may be appropriate. For example, in order to reduce hunger and promote good health and well-being (UN SDGs 2 and 3) analysis can be conducted by policymakers to understand ways in which local procurement can occur without (or with only minimally) increasing costs to schools. This may involve additional policy instruments that incentivize mainstream distributors to work with more local producers and processors. Similarly, creating opportunities for decent work and economic growth (UN SDG 8) may require more careful consideration around definitions of local so that benefits do not come at the expense of neighbouring locales. To do this, directly and indirectly impacted stakeholders (not just those within a particular political jurisdiction) can be included in conversations leading up to policy-decisions. In this context, policy instruments may include funding for programs that support technical assistance or supply chain infrastructure to engage regional farmers and ranchers. And, creating or growing markets for sustainably produced food (UN SDG 12) requires consideration of local conditions. Production systems associated with generating positive environmental impacts are not automatically transferable to other locations; their impacts may vary depending on geographical location. Accordingly, policy instruments that incentivize producer investment to achieve environmental benefits may be coupled with procurement incentives to achieve goals.

## Data availability statement

The original contributions presented in the study are included in the article/supplementary materials, further inquiries can be directed to the corresponding author.

## Author contributions

BJ, PM, SM, and KM contributed to conception and design of the study. BJ wrote the first draft of the manuscript. BJ and PM wrote sections of the manuscript. All authors contributed to the article and approved the submitted version.

## Funding

This research is supported by the Food Trails project, which received funding from the European Union’s Horizon 2020 Research and Innovation program, grant agreement no. 101000812, the National Institute of Food and Agriculture, the United States Department of Agriculture, under award no. 2021-68006-34029, and the US-UK Fulbright Commission.

## Conflict of interest

The authors declare that the research was conducted in the absence of any commercial or financial relationships that could be construed as a potential conflict of interest.

## Publisher’s note

All claims expressed in this article are solely those of the authors and do not necessarily represent those of their affiliated organizations, or those of the publisher, the editors and the reviewers. Any product that may be evaluated in this article, or claim that may be made by its manufacturer, is not guaranteed or endorsed by the publisher.
